# Abdominal attacks and treatment in hereditary angioedema with C1-inhibitor deficiency

**DOI:** 10.1186/1471-230X-14-71

**Published:** 2014-04-09

**Authors:** Eitan Rubinstein, Leslie E Stolz, Albert L Sheffer, Chris Stevens, Athos Bousvaros

**Affiliations:** 1 Division of Gastroenterology, Hepatology and Nutrition, Boston Children’s Hospital, 300 Longwood Ave, Fegan 5th Floor, Boston, MA 02115, USA; 2 Dyax Corp, Burlington, MA, USA; 3 Department of Allergy and Immunology, Brigham and Women’s Hospital, Boston, MA, USA

**Keywords:** Hereditary angioedema, Gastrointestinal angioedema, Ecallantide

## Abstract

**Background:**

Hereditary angioedema (HAE) is characterized by unpredictable attacks of debilitating subcutaneous and mucosal edema. Gastrointestinal attacks are painful, of sudden onset and often mistaken for acute abdomen leading to unnecessary surgery. The purpose of this study was to analyze symptom presentation of gastrointestinal angioedema in pediatric and adult HAE patients.

**Methods:**

Information collected during the clinical development of ecallantide for treatment of acute HAE attacks included affected anatomic location, accompanying symptoms, medical history, and pain assessments. Efficacy endpoints included Treatment Outcome Score (TOS, maximum score = 100; minimally important difference = 30), a point-in-time measure of treatment response, and time to treatment response.

**Results:**

Forty-nine percent of 521 HAE attacks only involved abdominal symptoms. The most commonly reported abdominal symptoms were distension (77%), cramping (73%) and nausea (67%). The most common pain descriptors were tender, tiring-exhausting, aching, cramping and sickening. White blood cell counts were elevated (>10 × 10^9^/L) in 23% of attacks (mean ± SD: 15.1 ± 11.27 × 10^9^/L). A high proportion of patients reported a history of abdominal surgery, including appendectomy (23%), cholecystectomy (16.4%), and hysterectomy (8.2%). Mean TOS at 4 hours post ecallantide was 77±33 versus 29±65 for placebo. Median time to significant symptom resolution was 165 minutes (95% CI 136, 167) for ecallantide versus >4 hours (95% CI 161, >4 hours) for placebo. Anaphylactic reactions occurred in 6 of the 149 treated patients.

**Conclusions:**

HAE should be considered in the differential diagnosis of patients with recurrent discrete episodes of severe, unexplained crampy abdominal pain associated with nausea.

**Trials registration:**

The data used in the analysis were gathered across multiple clinical trials conducted during the clinical development program for ecallantide. All of the studies were conducted using Good Clinical Practices (GCP) and in accordance with the ethical principles that have their origins in the Declaration of Helsinki. Each site that participated in the clinical trials obtained the appropriate IRB or Ethics Committee approval prior to enrolling any patients. All patients provided written informed consent prior to undergoing any study-related procedures. Pediatric patients provided written assent and their parents or guardians gave written informed consent.

The following trials have been registered at http://www.clinicaltrials.gov: EDEMA2 (identifier NCT01826916); EDEMA3 (identifier NCT00262080); EDEMA4 (identifier NCT00457015); and DX-88/19 (identifier NCT00456508).

## Background

Hereditary angioedema (HAE) is a rare and potentially life-threatening disease. It is characterized by non-pitting, non-pruritic swelling of subcutaneous or submucosal tissues of the skin, extremities, genitalia, respiratory and/or gastrointestinal tracts [1]. Because of the rarity of the disease and its self-resolving nature, many patients have historically faced a prolonged time to diagnosis (>10 years) [2]. The disease has an autosomal dominant pattern of inheritance and therefore patients typically present with a family history of angioedema. However, ~25% of cases are due to de novo mutations such that a family history is not always present [3]. Abdominal HAE attacks are very common, with up to 87% of patients rating abdominal attacks as excruciating or severely painful [4] often causing the patient to seek medical attention. Symptoms of abdominal HAE attacks often mimic other diseases such as appendicitis, small bowel obstruction, inflammatory bowel disease, gall bladder disease or diverticulitis. As a result, many patients report undergoing unnecessary abdominal surgery prior to diagnosis [5]. Thus, abdominal HAE attacks contribute significantly to the reduced quality of life and economic burden of HAE patients. Moreover, the accurate diagnosis of the disease, especially in patients who present only with recurrent abdominal symptoms, remains a challenge.

The primary cause of HAE is a deficiency in C1-inhibitor (HAE-C1INH), although HAE with normal C1-INH (HAEnC1) has been described [6]. C1-INH is a serine-protease inhibitor that regulates the activation of the coagulation, kallikrein-kinin and complement systems, with the kallikrein-kinin system playing a central role in the pathophysiology of HAE-C1INH [7]. During an HAE-C1INH attack, deficiency in C1-INH results in the unregulated activation of plasma kallikrein, a protease which cleaves high molecular weight kininogen to form the potent vasodilator bradykinin. As a result, excessive bradykinin is produced and activates the bradykinin B2 receptor on endothelial cells, causing the edema and inflammation characteristic of an HAE attack [7]. The precipitating events for an HAE attack are unknown but stress and minor trauma are thought to contribute.

The reported manifestations of intestinal angioedema in the literature include abdominal pain with or without nausea, vomiting and diarrhea, and abdominal distension from ascites [4,8]. These symptoms are due to edema of the bowel wall which can lead to partial or complete small bowel obstruction often with associated ascites [8,9]. Prior abdominal surgeries further complicate abdominal attacks due to the possibility of adhesions causing small bowel obstruction. To date, there have been limited prospective reports specifically detailing and quantitating the presenting symptoms of abdominal HAE attacks.

Herein, we provide prospectively collected data from adult and pediatric HAE-C1INH patients presenting with abdominal symptoms in the clinical program for ecallantide. Ecallantide is a subcutaneously administered plasma kallikrein inhibitor that was FDA approved in 2009 for the treatment of acute attacks of HAE in patients 16 years of age and older. These data provide information that should prove useful to the Gastroenterologist, Surgeon and ED Physician to consider HAE-C1INH when patients report with recurrent, unexplained abdominal distension, crampy abdominal pain with nausea.

## Methods

### Patient population

The analyses presented were generated from data collected in EDEMA0, EDEMA1®, EDEMA2®, EDEMA3®-DB, EDEMA3®-RD, EDEMA4® and DX-88/19 from patients with an HAE attack with any abdominal symptoms. All of the studies were conducted using Good Clinical Practices (GCP) and in accordance with the ethical principles that have their origins in the Declaration of Helsinki. Each site that participated in the clinical trials obtained the appropriate IRB or Ethics Committee approval prior to enrolling any patients (see Additional file 1 for list of IRB/IECs). All patients provided written informed consent prior to undergoing any study-related procedures. Pediatric patients provided written assent and their parents or guardians gave written informed consent.

Not all studies collected all of the same endpoints and therefore some of the analyses are based on different patient populations due to data availability. Eligible patients were 10 years of age or older (except in EDEMA0 in which patients were 18 years of age or older), with a diagnosis of Type I or Type II HAE. Patients had to report to the treatment center within 8 hours of an acute HAE attack that was moderate to severe in intensity. In DX-88/19 only, patients experiencing mild symptoms were also eligible for treatment at any time after the development of symptoms. Table 1 provides a summary of the endpoints collected in each study, along with the sample size (number of patients and attacks) analyzed.

**Table 1:** Data collected in EDEMA studies used for abdominal attacks analysis

	**EDEMA0**	**EDEMA1**	**EDEMA2**	**EDEMA3**	**EDEMA4**	**DX-88/19**	**Patients**	**Attacks**
							**(N)**	**(N)**
Symptoms at baseline	X	X	X^1^				112	296
Attack location			X^2^	X	X	X	149	521
McGill-SF pain questionnaire								
Pain descriptors	X	X	X^1^			X	242	502
Treatment outcomes			X^2^			X	111	386
VAS for pain			X^2^			X	111	386
Treatment outcome score			X^2^	X	X	X	149	521
Time to response				X	X	X	149	521
WBC count and surgical history	X	X	X^1^	X	X	X	183	569

*Abbreviations*: *SF* short form, *VAS* visual analog scale, *WBC* white blood cell.

^1^Includes all patients treated in EDEMA2.

^2^Only includes patients treated in the 30 mg subcutaneous ecallantide treatment arm.

### Treatments administered

In the EDEMA0, EDEMA1 and EDEMA2 trials, ecallantide was administered intravenously. During the final Phase II trial (EDEMA2), subcutaneous (SC) administration of 30 mg ecallantide was introduced and was used in all subsequent trials. Data describing the baseline attack characteristics (symptoms accompanying abdominal attacks, attack location, surgical history, white blood cell [WBC] count, McGill-Short Form [SF] Descriptor Severity Score) were generated from either IV treated patients alone or were pooled from both SC and IV treated patients, including some placebo-treated patients (as these data were collected prior to treatment, the route of administration has no bearing on the baseline attack characteristics). The data on the clinical response to ecallantide were generated only from those patients treated with SC ecallantide (the FDA-approved formulation).

### Abdominal attack characteristics

#### Symptoms at baseline and attack location

Baseline symptoms accompanying abdominal attacks were collected in 3 studies: EDEMA0, EDEMA1 and EDEMA2. In the SC arm of EDEMA2, as well as EDEMA3, EDEMA4 and DX-88/19 patients identified the location of their symptoms based on 5 symptom complexes: oropharyngeal head/neck (internal head/neck), gastrointestinal (GI)/abdominal, genital/buttocks, non-oropharyngeal head/neck (external head/neck), and cutaneous.

#### Short form McGill pain questionnaire

The McGill Pain Questionnaire (SF-MPQ) consists of 15 pain descriptors (11 sensory; 4 affective) which are rated on an intensity scale as 0 = none, 1 = mild, 2 = moderate or 3 = severe [10]. The pain descriptors are aggregated to calculate the sensory dimension score (includes the following pain descriptors: throbbing, shooting, stabbing, sharp, cramping, gnawing, hot-burning, aching, heavy, tender, and splitting) and the affective dimension score (includes the following descriptors: tiring-exhausting, sickening, fearful, cruel-punishing). Patients experiencing abdominal attacks were asked to complete the SF-MPQ at baseline and 4 hours post-treatment to better characterize the type of pain experienced during abdominal HAE attacks. The pain descriptor analysis includes data from 4 studies (EDEMA0, EDEMA1, EDEMA2 and DX-88/19) and includes all attacks with any abdominal symptoms. Descriptor severity scores were calculated by adding up the total score for all descriptors and dividing by number of attacks (N = 502). Data from the SC arm of EDEMA2 and DX-88/19 were used for calculating the treatment outcome in the sensory and affective dimension scores.

#### VAS pain

Patients who presented with HAE attacks with abdominal symptoms assessed their own perceptions of pain using a visual analog scale (VAS). Data from the SC arm of EDEMA2 and DX-88/19 were used for the VAS analysis. Patients were instructed to place a slash across a 100 mm long line at the position that best described their pain, with possible values ranging from 0 (no pain) to 100 (worst possible pain). Scoring took place at baseline and at 4 hours post-treatment.

### Efficacy assessments

#### Treatment outcome score (TOS)

Treatment outcome score (TOS) is a composite measure of symptom response to treatment. For each individual symptom complex, patients assess their change in symptom severity using a categorical scale weighted by the baseline symptom complex severity (significant improvement = 100, improvement = 50, same = 0, worsening = -50, significant worsening = -100) at 1, 2, 3, 4 and 24 hours post-treatment. Weighted scores across all sites are averaged to calculate TOS. A TOS value > 0 reflects improvement and the minimally important difference for the TOS was estimated to be 30.0 [11].

#### Time to response

To measure overall response to ecallantide or placebo, patients were asked to compare how they were feeling following treatment to how they were feeling before treatment. Patients were assessed every 15 min for the first 2 hours, every 30 min for hours 2 – 4, and then at 24 hours. Response measurements were made using a 5-category scale. Responses could be “a lot better or resolved”, “a little better”, “unchanged”, “a little worse” or “a lot worse”. Beginning of improvement was the first time within 4 hours the patient reported feeling “better” (i.e. either “a little better or a lot better or resolved”). The time to significant improvement was the first time within 4 hours the patient reported feeling “a lot better or resolved”.

### Safety assessments

Safety assessments included the collection of adverse events (AE) through final follow-up. An AE was defined as any untoward medical occurrence in a patient that received treatment that did not necessarily have a causal relationship with the treatment.

## Results

### Abdominal attack presentation

The individual symptoms associated with abdominal attacks at baseline were collected from patients in EDEMA0, EDEMA1 and EDEMA2. Table 2 provides a summary of these symptoms. The dataset includes 112 HAE patients treated for 296 HAE attacks; 154 of these attacks involved abdominal symptoms. The primary symptoms at baseline for attacks with abdominal symptoms (N = 154) were distension (77%), cramping (73%) and nausea (67%). Of note, diarrhea (14%) and vomiting (21%) were not predominant symptoms.

**Table 2:** **Symptoms associated with abdominal attacks**^**1**^

**Symptoms**	**n**^**2**^	**% all attacks (N = 296)**	**% attacks with any abdominal symptoms (N = 154)**
Distention	118	40%	77%
Cramping	112	38%	73%
Nausea	103	35%	67%
Vomiting	32	11%	21%
Diarrhea	21	7%	14%
Other symptoms^3^	62	21%	n/a

^1^All patients with abdominal symptoms in EDEMA0, EDEMA1 and EDEMA2.

^2^Number of attacks with the symptom at baseline.

^3^Free-form text box included: pain, discomfort, tenderness, hyperactive bowel sounds, increased bowl activity, constipation, urgency, peripheral swelling, rash, redness, itching.

**Figure 1:**
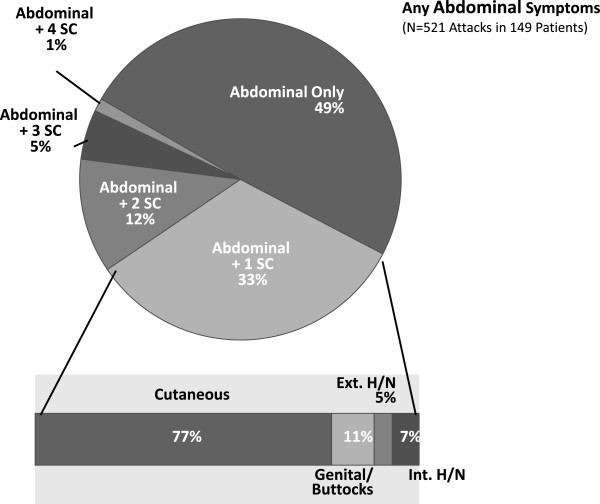
**Additional symptom complexes associated with all attacks with any abdominal symptoms treated with subcutaneous ecallantide.** All patients treated with 30 mg SC ecallantide in EDEMA2, EDEMA3, EDEMA4, and DX-88/19. Ext. = external, H/N = head/neck, Int. = internal, SC = subcutaneous. Percentages based on number of attacks.

Of all attacks with any abdominal pain (N = 521), 49% presented with isolated abdominal pain only and 33% presented with abdominal pain and symptoms at another location (Figure 1).

### Characteristics of abdominal pain in HAE patients

The character and severity of the pain associated with abdominal symptoms was captured using the SF-MPQ and VAS. Table 3 provides a summary of the SF-MPQ results ordered by the average severity score of the pain descriptor (scale: 0 = none; 3 = severe). The dataset included 502 attacks with any abdominal symptoms captured in EDEMA0, EDEMA1, EDEMA2 and DX-88/19.

**Table 3:** **McGill Descriptor Severity Score (N = 502 attacks)**^**1**^

**Pain descriptor**	**Total score**	**Average severity score**^**2**^
Tender	1020	2.03
Tiring-exhausting	912	1.82
Aching	892	1.78
Cramping	888	1.77
Sickening	834	1.66
Sharp	785	1.56
Heavy	778	1.55
Stabbing	629	1.25
Gnawing	628	1.25
Shooting	595	1.19
Throbbing	592	1.18
Punishing-cruel	492	0.98
Hot-burning	397	0.79
Splitting	384	0.76
Fearful	346	0.69

^1^All patients with abdominal symptoms in EDEMA0, EDEMA1, EDEMA2 and DX-88/19.

^2^Intensity scale: 0 = none, 3 = severe.

**Figure 2:**
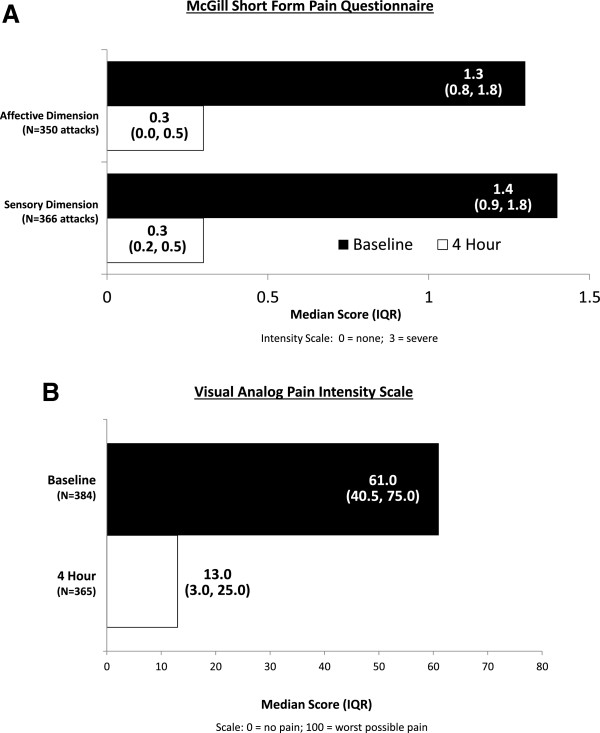
**Changes in pain during an HAE attack at 4 hours post-dosing with 30 mg SC ecallantide. A)** McGill Pain Score in both the affective and sensory dimensions at baseline and 4 hours post-dosing with ecallantide. **B)** Visual Analog Scale pain intensity score at baseline and 4 hours post-dosing with ecallantide. Data from both analyses includes all patients treated with 30 mg SC ecallantide in EDEMA2 and DX-88/19 with any abdominal symptoms; analysis excludes attacks with missing data.

The severity of the affective and sensory dimensions of the McGill pain score at baseline and 4 hours post-dosing with 30 mg SC ecallantide is presented in Figure 2A. Treatment with ecallantide reduced the pain severity score (median [SD]) at baseline to 4 hours post-dosing from 1.3 (0.8, 1.8) to 0.3 (0.0, 0.5) in the affective dimension (N = 350 attacks) and from 1.4 (0.9, 1.8) to 0.3 (0.2, 0.5) in the sensory dimension (N = 366 attacks). Similar to the findings using the SF-MPQ, ecallantide also reduced median pain intensity as measured using the VAS (Figure 2B).

### White blood cell count and surgical history

White blood cell (WBC) count was measured in all HAE attack patients. This dataset of primary abdominal attacks included 183 patients with 569 primary abdominal attacks; defined as moderate or severe abdominal symptoms in the absence of laryngeal symptoms. In 132 of these attacks, WBC counts were elevated (mean ± SD: 15.1 ± 11.27 × 10^9^/L; elevated = > 10 × 10^9^/L). An elevated WBC count was measured with 64% of severe abdominal attacks compared to 34% of moderate attacks. In these 183 patients, 23% had previously received an appendectomy, 16.4% had received a cholecystectomy, 1.6% had received a bowel resection, 2.7% had an exploratory laparotomy, and 8.2% had a hysterectomy.

### Characteristics of abdominal pain in pediatric patients

Abdominal symptoms in the pediatric subgroup of patients were evaluated to determine if children differed from adults in their abdominal HAE attack presentation. For this pediatric subgroup, data from EDEMA0, EDEMA1, EDEMA2 and DX-88/19 were analyzed. The pediatric subgroup included 29 patients with 72 attacks (age 9–17 years; 9 year old patient obtained a waiver for inclusion in the study). Forty of 72 (56%) attacks were classified as primary abdominal attacks and 28 of 72 (40%) attacks were abdominal attacks only. As with adults, primary abdominal attacks were defined as moderate or severe abdominal symptoms in the absence of laryngeal symptoms. McGill data at baseline was collected for 50 attacks in 19 pediatric patients with any abdominal symptoms. The top 5 descriptors of abdominal pain were tender (1.92), cramping (1.64), aching (1.58), sickening (1.50) and tiring-exhausting (1.44). VAS pain data was available from 15 pediatric patients treated for 39 attacks. At baseline, the median (IQR) VAS pain score was 62 (50, 77) and 4 hours post-ecallantide dosing this was reduced to a median score of 11 (2, 25). The McGill descriptors and VAS pain scores in pediatric patients are numerically similar to those reported above for the overall population.

### Clinical response of HAE attacks with abdominal symptoms to SC ecallantide

#### Patient demographics

Table 4 provides summary data of patient demographics and the number of attacks with any abdominal symptoms treated with 30 mg SC ecallantide in EDEMA2, EDEMA3, EDEMA4 and DX-88/19; N = 149 patients for 521 attacks. The overall population of SC treated patients included 230 patients with 929 attacks and therefore 65% (149/230) of patients presented with HAE attacks with abdominal symptoms. The average number of attacks with abdominal symptoms per patient was 3.5 (4.0 SD; range 1–28).

**Table 4:** **Patient demographics and number of abdominal attacks in patients treated with 30 mg subcutaneous ecallantide**^**1**^

	**Patients (N = 149)**
**Patient demographics**	
Number of attacks	521
Age, mean (range)	34.4 (10–77)
Gender, %	
Female	70.5
Male	29.5
Race/Ethnicity, %	
Caucasian	88.6
Black	4.7
Hispanic	4.7
Other	2.0
**Number of abdominal attacks per patient**	
Mean (SD)	3.5 (4.0)
Range	1-28
Total Attacks, n (%)	
1 attack	57 (38)
2 attacks	29 (19)
3 attacks	24 (16)
4 attacks	8 (5)
5 attacks	6 (4)
>5 attacks	25 (17)

^1^All patients treated with 30 mg subcutaneous ecallantide in EDEMA2, EDEMA3, EDEMA4 and DX-88/19 with any abdominal symptoms.

#### Treatment outcome score

**Figure 3:**
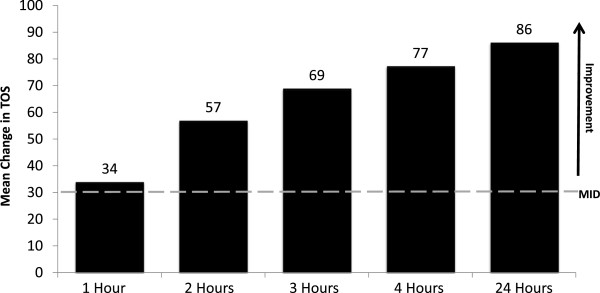
**Treatment Outcome Score (TOS) in all HAE attacks with any abdominal symptoms treated with SC ecallantide.** Includes all patients treated with 30 mg SC ecallantide in EDEMA2, EDEMA3, EDEMA4, and DX-88/19 (N = 521 attacks in 149 patients); analysis excludes attacks with missing data.

Figure 3 shows the mean change in Treatment Outcome Score (TOS) at 1, 2, 3, 4 and 24 hours following SC ecallantide for all HAE attacks with any abdominal symptoms. By 1 hour following SC ecallantide administration, patients reached the minimally important difference (MID = 30) for the TOS, which reflects a clinically meaningful treatment response. As a comparison, MacGinnitie et al. reported a TOS at 4 hours of 29 in placebo-treated patients with abdominal symptoms [12].

#### Time to response

The time to beginning of improvement and the time to significant improvement were collected in patients with HAE attacks with any abdominal symptoms. The median time (95% CI) to beginning of improvement was 54 minutes (52, 55) following SC ecallantide administration versus 114 minutes in placebo treated patients. The median time (95% CI) to significant symptom resolution was 165 minutes (136, 167) for ecallantide treated attacks versus > 4 hours (161, > 4 hours) for placebo.

#### Safety

The safety profile of ecallantide was similar to previous reports, with potentially serious hypersensitivity representing the main safety concern. Anaphylaxis (based on NIAID criteria [13]) occurred in 6 of the 149 patients treated with SC ecallantide. All 6 reactions resolved following standard treatment (including epinephrine in 2 patients and no treatment in 2 patients). The most common treatment-emergent adverse events in all patients with any abdominal symptoms treated with SC ecallantide (N = 149) were: headache (14%), nausea (9%), fatigue (8%) and HAE (7%).

## Discussion

Diagnosis of HAE-C1INH in patients with recurrent abdominal pain can be challenging, especially in the approximately 50% of cases that present only with abdominal symptoms. These patients are very likely to be referred to a Gastroenterologist at some point, particularly given that abdominal symptoms occur in approximately 80% of patients [14] and on average it takes 10 years from the time of onset of symptoms until a diagnosis is made [2]. It is commonplace for HAE-mediated abdominal pain to be mistaken for other causes of abdominal pain, such as appendicitis, cholecystitis and small bowel obstruction [15,16]. As a consequence, many HAE patients report having undergone unnecessary surgeries for their HAE symptoms including exploratory laparotomy, cholecystectomy and hysterectomy [15,17]. We observed a similar surgical history in patients enrolled in the clinical development program for ecallantide. These results highlight the difficulty in initially suspecting and then diagnosing HAE. Once a diagnosis of HAE is suspected, there are several published guidelines/consensus documents to aid the clinician in the diagnosis and management of HAE patients [18-21].

Historical data suggests that up to 80% of HAE patients have recurrent abdominal pain [22]. This percentage is similar to that observed in the clinical development program of ecallantide in which 65% (149/230) of patients treated with SC ecallantide presented with HAE attacks with abdominal symptoms. The most commonly reported symptoms associated with abdominal attacks in this dataset were distension (77%), cramping (73%) and nausea (67%), which is in agreement with previous reports [4,23]. These same symptoms were also commonly reported in pediatric patients with HAE.

The percentage of HAE attacks with symptoms of diarrhea (14%) and vomiting (21%) reported during the ecallantide development program were lower than those reported in previous studies. In two retrospective reports from Bork and colleagues, the percentage of attacks with vomiting were 83.3% and 73.3%, while 41.8% and 40.6% of attacks had symptoms of diarrhea [4,23]. The reason for the discrepancies between these studies is unclear but may be due to differences in the severity of attacks included in the analyses, as well as how the severity of attacks was defined. Both reports from Bork et al. included a high percentage of severe attacks, 78.7% and 70.0% respectively. In contrast, in the ecallantide development program 37.9% of all attacks with abdominal symptoms were rated as severe. It may be that diarrhea occurs as the attack is resolving and the timing of the data collection in the EDEMA program (within 8 hours of symptom onset vs an average attack duration of 2 to 5 days) may not have detected this later symptom. In addition, retrospective analyses are subject to recall bias which may also contribute to the differences observed between studies.

White blood cell (WBC) count is often used in the differential diagnosis of acute abdominal pain and was collected in patients with primary abdominal attacks in the ecallantide development program. Interestingly, WBC count was elevated in ~25% of attacks and was more likely to be elevated in attacks rated as severe. This finding is in agreement with several published case reports of abdominal HAE attacks and therefore suggests that elevated WBC count is not a reliable measure to include or exclude HAE in the differential diagnosis of abdominal pain [24,25]. Indeed, Ohsawa et al. recently reported significant leukocytosis with neutrophilia, as well as increased levels of hematocrit and low C-reactive protein (CRP) in HAE patients with severe abdominal pain, when compared with laboratory values when patients were not experiencing an attack [26].

To date, there is no reliable marker to define abdominal angioedema upon presentation of symptoms. Even in patients with a confirmed diagnosis of HAE, a diagnosis of an abdominal attack depends upon clinical judgment and the demonstrated efficacy of specific treatments. In undiagnosed patients, once HAE is suspected, blood tests can be used to confirm the diagnosis but these results will not be available for diagnosis and treatment in the initial emergency setting. The initial screening test is C4 blood levels, which will be low in HAE patients at baseline and even lower during an acute attack. However, a reduced C4 level during an attack does not identify the presence or absence of angioedema. The C4 test is inexpensive, common to most hospital laboratories, and typically has a high sensitivity and specificity, with at least 95% of HAE-C1INH patients showing a reduced C4 level during remission and virtually 100% showing reduced C4 during an attack [27]. If the C4 is low, an abnormal functional C1-INH test would confirm the diagnosis of HAE [27]. The endoscopic and computerized tomography (CT) appearance of abdominal angioedema has been previously reported and although nonspecific, in the right clinical setting of association with skin swelling and a family history, can suggest the diagnosis [16,24,25,28]. Typical observations associated with abdominal angioedema include small bowel mucosal thickening, thumb printing of the bowel wall and ascites [16,25].

There are now several therapeutic options available for the treatment of HAE. These include both long-term prophylaxis, as well as the treatment of acute attacks but studies of comparative effectiveness among treatments have not been conducted. Attenuated androgens have long been used for HAE prophylaxis but can have serious side-effects [29,30]. CINRYZE® (ViroPharma, Exton, PA) is a nanofiltered, C1-INH (Human) replacement product also approved for prophylaxis [31]. Acute treatment options include: Berinert® (CSL Behring, Marburg, Germany), a nanofiltered C1-INH (Human) replacement product [32]; FIRAZYR® (icatibant, Shire, Lexington, MA), a bradykinin B2 receptor antagonist [33]; and KALBITOR® (ecallantide, Dyax Corp., Burlington, MA), a plasma kallikrein inhibitor [34].

Each of the HAE therapies have demonstrated efficacy in the management and treatment of HAE but also carry a risk of harm. As stated in the package inserts and reported in the literature, the use of C1 Esterase Inhibitor (Human) products is associated with serious arterial and venous thromboembolic events [31,32,35]. The use of icatibant is associated with injection site reactions, which occurred in almost all patients (97%) in clinical trials [33]. The major risk associated with use of ecallantide is hypersensitivity reactions [34]. Anaphylaxis has been reported after administration of ecallantide and because of this risk, should only be administered by a healthcare professional with appropriate medical support to manage anaphylaxis and HAE [34]. In the study presented here, ecallantide was shown to elicit a clinically meaningful treatment response for abdominal attacks within 1 hour post dosing, with significant symptom resolution occurring in 2 to 4 hours. Thus, in patients diagnosed with HAE-C1INH, a variety of effective treatment options are available to minimize the symptoms of HAE attacks and improve overall quality of life.

## Conclusion

In summary, patients that present with attacks of recurrent abdominal pain that completely resolve in the absence of other symptoms are challenging to diagnose. The data from this study provide information that should aid the Gastroenterologist in suspecting the diagnosis of HAE in patients with such unexplained bouts of severe abdominal pain. Patients with HAE are likely to have intermittent exacerbations of moderate to severe, poorly localized pain, described as tender, tiring-or exhausting, aching cramping or sickening. Elevation of white blood cells frequently occurs during severe abdominal attacks and may lead to exploratory laparotomy for concern of other more common conditions. As Gastroenterologists increase their awareness of HAE, patients will be diagnosed earlier to receive both timely and effective treatment.

## Competing interests

Dyax Corp designed and provided funding for the studies described in this article. Dr. Rubinstein has no conflicts to declare. Dr. Bousvaros reports personal fees from Dyax Corp, personal fees from Millennium pharmaceuticals, grants and personal fees from Merck pharmaceuticals, personal fees from Up to Date, personal fees from Imedex, non-financial support from Prometheus, non-financial support from Nutricia, personal fees from Cubist, outside the submitted work. Dr. Sheffer reports serving as a principle investigator for Dyax Corp. Leslie Stolz, PhD and Joseph Biedenkapp, PhD are full-time employees of Dyax Corp. Chris Stevens, MD is a consultant for Dyax Corp. The authors declare that they have no competing interests.

## Authors’ contributions

ER participated in the analysis and interpretation of data and reviewed and revised the article; LS participated in the conception and design, analysis and interpretation of data and reviewed and revised the article; AS participated in the conception and design, analysis and interpretation of data and reviewed and revised the article; CS participated in the conception and design, analysis and interpretation of data and reviewed and revised the article; AB participated in the analysis and interpretation of data and reviewed and revised the article; all authors approved the final article as submitted.

## Pre-publication history

The pre-publication history for this paper can be accessed here:

http://www.biomedcentral.com/1471-230X/14/71/prepub

## Supplementary Material

Additional file 1 List of IRB/IECs in the clinical development program for ecallantide. Click here for file

## cknowledgements

The authors wish to thank Joseph Biedenkapp, PhD (Dyax Corp.) for his assistance with the drafting of this manuscript.
