# Supplementary material for: Abdominal attacks and treatment in hereditary angioedema with C1-inhibitor deficiency
Source: BMC Gastroenterol. 2014 Apr 9;14:71. doi: 10.1186/1471-230X-14-71 (PMC4101849; doi:10.1186/1471-230X-14-71)
Supplement: Additional file 1 — List of IRB/IECs in the clinical development program for ecallantide. [file 1471-230X-14-71-S1.docx]

**List of IRB/IECs in the clinical development program for ecallantide**

**EDEMA0**

Site 1: Investigator - Konrad Bork, MD

Institutional Ethics Committee

Ethik-Kommission der Landesarztekammer Rheinland-Pfalz

Chairman: Hoffart, MD

Site 2: Investigator - Marco Cicardi, MD

Institutional Ethics Committee

Ospedale Maggiore Di Milano I.R.C.C.S.

President of the Committee: Prof. Antonio Randazzo

Site 3: Investigator: Teresa Cabellero, MD, PhD

Ethics Committee

Hospital Universitario La Paz

Chairman: Antonio Gil Aguado

Site 4: Investigator: Teresa Gonzalez-Quevedo

Ethics Committee

Hospitales Universitarios Virgen del Rocio

President: Ana M Casa Fernandex de Tejerina

Site 5: Investigator: Hilary Longhurst, MD

Ethics Committee

East London and the City Research Ethics Committee

Chairman: Richard Smith

**EDEMA1®**

Site 01: Investigator - Suresh Anne, MD

Institutional Review Board

Hurley Medical Center

Chairman: Robert Lavoie, PharmD

Site 02: Investigator - Thomas Prescott Atkinson, MD, PhD

Institutional Review Board

University of Alabama Birmingham

Chairman: Ferdinand Urthaler, MD

Site 03: Investigator - Timothy J. Craig, DO

Institutional Review Board

Milton S. Hershey Medical Center

Chairman: Kevin Gleeson, MD

Site 04: Investigator - Paula J. Busse, MD

Institutional Review Board

Mount Sinai Board of Medicine

Chairman: Jeffrey H. Silverstein, MD

Site 05: Investigator - Curtis Ray Evans, MD

Institutional Review Board

Harris Methodist Fort Worth Hospital/Southwest

Chairman: Kathleen Crowley, MD

Site 07: Investigator - John Andrew Grant, MD

Institutional Review Board

University of Texas Medical Brandh

Chairman IRB No. 1: Frank C. Schmalstieg, MD

Chairman IRB No. 2: Joseph B. Zwischenberger, MD

Site 09: Investigator - David H. Irwin, MD

Institutional Review Board

Alta Bates Summit Medical Center

Co-chairs: Joanna Cooper MD, James McFeely MD,

Site 10: Investigator - Kraig Warren Jacobson, MD

Institutional Review Board

Western Institutional Review Board

Chairman: William C. Jacobs, BA

Site 12: Investigator - Richard F. Lockey, MD

Institutional Review Board

University of Southern Florida

Chairman: Barry B. Bercu, MD

Site 13: Investigator - William R. Lumry, MD

Institutional Review Board

Presbyterian Hospital of Dallas

Chairman: James F. Strauss, MD

Site 14: Investigator - James Edward Maher, MD

Institutional Review Board

Baptist Hospital, Pensacola Florida

Chairman: Paula Montgomery, MD

Site 15: Investigator - Hobert L. Pence, MD

Institutional Review Board

Western Institutional Review Board

Chairman: Theodore D. Schultz, JD

Site 17: Investigator - Lynda Schneider, MD

Institutional Review Board

Children's Hospital Boston

IRB Administrator: Jillian Richard

Site 18: Investigator - Aziza Tahir Shad, MD

Institutional Review Board

Georgetown University

Chairman: Mary Young, MD

Site 19: Investigator - Vincent R. Bonagura, MD

Institutional Review Board

Biomedical Research Alliance of New York, LLC

Chairman: Keith Krasinski, MD

Site 20: Investigator - Hassan N Taki, MD

Institutional Review Board

Western International Review Board

Chairman: William C. Jacobs, BA

Site 21: Investigator - Dale T. Umetsu, MD, PhD

Administrative Panel for Human Subjects in Clinical Research

Stanford Univeristy

Chairman: Donald R. Stanski, MD

Site 22: Investigator - Arthur B. Vegh, MD

Institutional Review Board

Multicare Health System

Chairman: Richard E. Shine, Pharm D

Site 23: Investigator - Robert W. Wilkinson, MD

Institutional Review Board

Hawaii Pacific Health

Chairman: Raul Rudoy, MD

Site 24: Investigator - Henry Li, MD, PhD

Institutional Review Board

Western Institutional Review Board

Chairman: William C. Jacobs, BA

Site 25: Investigator - Bruce L. Zuraw, MD

Institutional Review Board

ScrippsHealth Scripps Green Hospital

Name of Chairman on file with Office for Human Research Protections (OHRP) under IRB 000001283.

Site 27: Investigator - Arye Rubinstein, MD, PhD

Institutional Review Board

Biomedical Research Alliance of New York, LLC

Chairman: Keith Kransinki, MD

Site 28: Investigator - Arye Rubinstein, MD, PhD

Institutional Review Board

Biomedical Research Alliance of New York, LLC

Chairman: Keith Kransinki, MD

Site 29: Investigator - Jose Venzor III, MD

Institutional Review Board

Western Institutional Review Board

Chairman: Theodore D. Schultz, JD

Site 31: Investigator - Shmuel Kivity, MD

Institutional Review Board

Tel Aviv Sourasky Medical Center

Chairman: Shmuel Kivity, MD

Site 32: Investigator - Paul de Buysscher, MD

Institutional Ethics Committee

Elisabeth Ziekenhuis

Chairman: C. Roels, MD

**EDEMA2®**

Site 03

Timothy Craig, DO

Penn State College of Medicine

Institutional Review Board/Human Subjects Protection Office

500 University Drive, H112

Hershey, PA 17033

Chair: Kevin Gleeson, MD

Site 07

J. Andrew Grant, MD

Institutional Review Board

University of Texas Medical Branch

301 University Blvd.

Gail Borden Bldg. 2.224

Galveston, TX 77555-0673

Chairs: Frank C. Schmalstieg Jr., MD, PhD; Joseph B. Zwischenberger, MD

Site 09

David Irwin, MD

Alta Bates Summit Institutional Review Board

2450 Ashby Avenue

Berkeley, CA 94705

Chairs: Claire Borkert; James McFeely

Site 12

Richard Lockey, MD

USF Office of Research, Division of Research Compliance

Institutional Review Boards, MPA no. FWA00001669

12901 Bruce B. Downs Blvd.

Tampa, FL 33612

Chair: Barry B. Bercu, MD

Site 13

William R.Lumry, MD

Western Institutional Review Board

3535 Seventh Ave., SW

Olympia, WA 98502

Chair: Theodore D. Schultz

Site 15

Hobert L. Pence, MD

Western Institutional Review Board

3535 Seventh Ave., SW

Olympia, WA 98502-5010

Chair: Theodore D. Schultz

Site 17

Lynda Schneider, MD

Committee on Clinical Investigation

Children’s Hospital of Boston

300 Longwood Ave.

Boston, MA 02115

Chair: not available

Site 18

Aziza Shad, MD

MedStar Research Institute-Georgetown University Oncology Institution Review Board

Med/Dent Building, SW 104

3900 Reservoir Road, NW

Washington, DC 20057-2197

Chair: David J. Perry, MD

Site 19

Vincent Bonagura, MD

Biomedical Research Alliance of New York, LLC

Institutional Review Board

225 Community Drive

Suite 100

Great Neck, New York 11021

Chair: Keith Krasinski

Site 22

Arthur Vegh, MD

Western Institutional Review Board

3535 Seventh Avenue SW

Olympia, WW 98502-5010

Chair: Theodore D. Schultz

Site 23

Robert Wilkinson, MD

Hawaii Pacific Health Institutional Review Board

100 Ward Avenue, Suite 1045

Honolulu, HI 96814

Chair: Raul Rudoy, MD

Site 24

Huamin (Henry) Li, MD, PhD

Western Institutional Review Board

3535 Seventh Ave., SW

Olympia, WA 98502-5010

Chair: Theodore D. Schultz

Site 27 and Site 28

Arye Rubinstein, MD

Biomedical Research Alliance of New York, LLC

Institutional Review Board

225 Community Drive

Suite 100

Great Neck, New York 11021

Chair: Keith Krasinski

Site 29

Jose Venzor III, MD

Western Institutional Review Board

3535 Seventh Avenue SW

Olympia, WW 98502-5010

Chair: Theodore D. Schultz

Site 50

Gregory Marcotte, MD

Christiana Care Corporation

Institutional Review Board

501 West 14 Street

P.O. Box 1668

Wilmington, DE 19899

Chair: not available

Site 51

Jeffrey G.Leflein, MD

St. Joseph Mercy Health System Clinical Research Committee

5301 East Huron River Drive

P.O. Box 995

Ann Arbor, MI 48106-0995

Chair: James Mitchiner, MD

Site 52

I. Leonard Bernstein, MD

University of Cincinnati

Institutional Review Board

G08 Wherry Hall

Eden and Albert Sabin Way

Cincinnati, Ohio 452687-0567

Chair: Michael J. Linke, PhD

Site 53

Mark Alan Davis-Lorton, MD

Western Institutional Review Board

3535 Seventh Avenue SW

Olympia, WA 98508-2029

Chair: Theodore D. Schultz

Site 54

Stacie M. Jones, MD

University of Arkansas for Medical Sciences Institutional Review Board

4301 W. Markham Street, Slot 636

Little Rock, AR 72205-7199

Chair: Jimmie Valentine

Site 55

Mary C. Tobin, MD

Office of Research Affairs

Rush University Medical Center

439 Professional Building

1725 West Harrison St.

Chicago, IL 60612

Chair: Allen Korenblit

Site 56

William H. Yang, MD

British Columbia Institutional Review Board

Institutional Review Board Services

14845-6 Yonge St, Suite 328

Aurora, Ontario, Canada

L4G 6H8

Chair: George Price, MD

Site 57

Bruce Ritchie, MD

Health Research Ethics Board

Judith Abbott, Administrative Coordinator

University of Alberta, 213 HMRC

8440 112 Street

Edmonton, AB, Canada

T6G 2S2

Chair: not available

Site 58

Waldon, G. Bruce MD

Mercy Health System Northwest Arkansas IRB#1

1200 West Walnut

Rogers, AR 72756

Chair: Diane Brown, RN

Site 59

Wheatley, Lisa M. MD

Western Institutional Review Board

P.O. Box 12029

Olympia, WW 98508-2029

Chair: Theodore D. Schultz

Site 60

Russell, Donald, MD

McCann, William A., MD

Western Institutional Review Board

3535 Seventh Ave., SW

Olympia, WA 98502-5010

Chair: Theodore D. Schultz

Site 61

Ronald Ferdman, MD

Committee on Clinical Investigations

Children's Hospital of Los Angeles

4650 Sunset Boulevard MS#23

Los Angeles, CA 90027

Chair: Thomas G. Keens, MD

Site 62

Karl V. Sitz, MD

Western Institutional Review Board

3535 Seventh Avenue SW

Olympia, WW 98502-5010

Chair: Theodore D. Schultz

Site 63

Amy M. Sprague, MD

Sterling IRB

6300 Powers Ferry Road

Suite 600-351

Atlanta, GA 30339

Chair: Sally P. Green, MD

Site 64

Jacques Hébert, MD

IRB

45 Wellington Street East

Aurora, Ontario, Canada

L4G 1H6

Chair: George Price, MD

Site 65

Albert L. Sheffer, MD

Partners human Research Committee

116 Huntington Avenue

Suite 1002

Boston, MA 02116

Chair: not available

**EDEMA3®-DB**

Site 301

L. Henry Li, MD

Western Institutional Review Board

3535 Seventh Avenue, SW—Olympia, WA 98502-5010

PO Box 12029—Olympia, WA 98508-2029

Chair: Theodore D. Schultz, JD

Site 302

David Hurewitz, MD

Western Institutional Review Board

Western Institutional Review Board

3535 Seventh Avenue, SW—Olympia, WA 98502-5010

PO Box 12029—Olympia, WA 98508-2029

Chair: Theodore D. Schultz, JD

Site 303

William Lumry, MD

Western Institutional Review Board

3535 Seventh Avenue, SW—Olympia, WA 98502-5010

PO Box 12029—Olympia, WA 98508-2029

Chair: Theodore D. Schultz, JD

Site 304

Don McNeil, MD

Western Institutional Review Board

3535 Seventh Avenue, SW—Olympia, WA 98502-5010

PO Box 12029—Olympia, WA 98508-2029

Chair: Theodore D. Schultz, JD

Site 305

Jeffrey Leflein, MD

St. Joseph Mercy Health System Clinical Research Committee

5301 East Huron River Drive

PO Box 995 Ann Arbor, MI 48106-0995

Chair: James Mitchiner, MD

Site 306

Lisa Wheatley

Western Institutional Review Board

3535 Seventh Avenue, SW—Olympia, WA 98502-5010

PO Box 12029—Olympia, WA 98508-2029

Chair: Theodore D. Schultz, JD

Site 307

William McCann, MD

Donald Russell, MD

Western Institutional Review Board

3535 Seventh Avenue, SW—Olympia, WA 98502-5010

PO Box 12029—Olympia, WA 98508-2029

Chair: Theodore D. Schultz, JD

Site 308

Stacie Jones, MD

UAMS Institutional Review Board

4301 W. Markham St., Slot 636, Little Rock, AR 72205

Chair: Jimmie Valentine, PhD

Site 309

Karl Sitz, MD

Western Institutional Review Board

3535 Seventh Avenue, SW—Olympia, WA 98502-5010

PO Box 12029—Olympia, WA 98508-2029

Chair: Theodore D. Schultz, JD

Site 310

Timothy Craig, MD

Penn State College of Medicine,

Milton S. Hershey Medical Center

500 University Drive, PO Box 850, Hershey, PA 17033

Chair: Kevin Gleeson, MD

Site 311

Mark Neustrom, MD

Western Institutional Review Board

3535 Seventh Avenue, SW—Olympia, WA 98502-5010

PO Box 12029—Olympia, WA 98508-2029

Chair: Theodore D. Schultz, JD

Site 312

Vincent Bonagura, MD

Biomedical Research Alliance of New York, LLC

Institutional Review Board

225 Community Drive, Suite 100, Great Neck, NY 11021

Chair: Keith Krasinski, MD

Site 313

Mark Davis-Lorton, MD

Western Institutional Review Board

3535 Seventh Avenue, SW—Olympia, WA 98502-5010

PO Box 12029—Olympia, WA 98508-2029

Chair: Theodore D. Schultz, JD

Site 314

Usha Sunkara, MD

Alta Bates Institutional Review Board

2450 Ashby Ave., Berkeley, CA 94705

Chairs: Claire Borkert, MD; James McFeely, MD

Site 315

Arthur Vegh, MD

Western Institutional Review Board

3535 Seventh Avenue, SW—Olympia, WA 98502-5010

PO Box 12029—Olympia, WA 98508-2029

Chair: Theodore D. Schultz, JD

Site 316

Gregory Marcotte, MD

Christiana Care Institutional Review Board

501 West 14th Street, Wilmington, DE 19899

PO Box 1668, Wilmington, DE 19899

Chairs: Robert Cox, MD; Gary Johnson, PhD

Site 317

Albert Sheffer, MD

Brigham's and Women's Hospital Institutional Review Board

Partners Human Research Committee

116 Huntington Ave., Suite 1002, Boston, MA 02115

Site 318

William Yang, MD

Ontario Institutional Review Board

14845-6 Yonge Street, Suite 328, Aurora, ON, Canada L4G 6H8

Chair: Allan Knight

Site 319

Robert Wilkinson, MD

Hawaii Pacific Health Institutional Review board

1100 Ward Avenue, Suite 1045, Honolulu, HI 96814

Chair: not available

Site 320

Duane Wombolt, MD

Western Institutional Review Board

3535 Seventh Avenue, SW—Olympia, WA 98502-5010

PO Box 12029—Olympia, WA 98508-2029

Chair: Theodore D. Schultz, JD

Site 321

Jacques Hebert, MD

IRB Services

45 Wellington St. East, Aurora, Ontario, Canada L4G 1H6

Chair: not available

Site 322

Robert Chrzanowski

Western Institutional Review Board

3535 Seventh Avenue, SW—Olympia, WA 98502-5010

PO Box 12029—Olympia, WA 98508-2029

Chair: Theodore D. Schultz, JD

Site 323

Richard Lockey, MD

Western Institutional Review Board

3535 Seventh Avenue, SW—Olympia, WA 98502-5010

PO Box 12029—Olympia, WA 98508-2029

Chair: Theodore D. Schultz, JD

Site 324

Sai Karlapundi, MD

Western Institutional Review Board

3535 Seventh Avenue, SW—Olympia, WA 98502-5010

PO Box 12029—Olympia, WA 98508-2029

Chair: Theodore D. Schultz, JD

Site 325

Neil Wallen, MD

Western Institutional Review Board

3535 Seventh Avenue, SW—Olympia, WA 98502-5010

PO Box 12029—Olympia, WA 98508-2029

Chair: Theodore D. Schultz, JD

Site 326

Robyn Levy, MD

Western Institutional Review Board

3535 Seventh Avenue, SW—Olympia, WA 98502-5010

PO Box 12029—Olympia, WA 98508-2029

Chair: Theodore D. Schultz, JD

Site 327

Mary Tobin, MD

Rush University Medical Center Institutional Review Board

1725 W. Harrison, Professional Building II, Suite 439, Chicago, IL 60612

Chairs: Allen Korenblit, MD; Howard Kravitz, DO, MPH

Site 328

Gary Kleiner, MD, PhD

Western Institutional Review Board

3535 Seventh Avenue, SW—Olympia, WA 98502-5010

PO Box 12029—Olympia, WA 98508-2029

Chair: Theodore D. Schultz, JD

Site 329

David Elkayam, MD

Western Institutional Review Board

3535 Seventh Avenue, SW—Olympia, WA 98502-5010

PO Box 12029—Olympia, WA 98508-2029

Chair: Theodore D. Schultz, JD

Site 331

Aziza Shad, MD

Georgetown University Institutional Review Board

SW 104 Medical Dental Building

3900 Reservoir Rd. NW, Washington, DC 20057

Chair: Mary Young, MD

Site 332

Arye Rubenstein, MD

Biomedical Research Alliance of New York, LLC

Institutional Review Board

225 Community Drive, Suite 100, Great Neck, NY 11021

Chair Keith Kransinski, MD

Site 333

Eric Schenkel, MD

Western Institutional Review Board

3535 Seventh Avenue, SW—Olympia, WA 98502-5010

PO Box 12029—Olympia, WA 98508-2029

Chair: Theodore D. Schultz, JD

Site 334

David Huston

Institutional Review Board

Baylor College of Medicine

One Baylor Plaza, Suite 600D, Houston, TX 77030

Chair: not available

Site 335

James Baker, MD

Western Institutional Review Board

3535 Seventh Avenue, SW—Olympia, WA 98502-5010

PO Box 12029—Olympia, WA 98508-2029

Chair: Theodore D. Schultz, JD

Site 337

Andrew MacGinnitie, MD

University of Pittsburgh Institutional Review Board

3500 Fifth Avenue, Ground Level, Pittsburgh, PA 15213

Chair: Richard Guido, MD

Site 360

Paul de Buysscher, MD, PhD

Ethische Comimissie - Campus Eeklo

vzw 'Elisabeth Zeikenhuis - Heilig Hartkliniek'

Moeie 18 - 9900 Eeklo, Belgium

Chair: Vincent Vandevelde

Site 361

Shmuel Kivity, MSc, MD

Helsinki Committee

Tel Aviv Sourasky Medical Center

Sackler Faculty of Medicine, Tel Aviv University

6 Weizman St.

Tel Aviv 64239, Israel

Chair: not available

Site 365

Marco Cicardi, MD

Comitato Etico Indipendente dell'Ospedale San Giuseppe

Ordine ospedaliero di San Giovanni de DIO

Fatebenefratelli di Milano

Via San Vittore, 12

20123, Milan, Italy

Chair: not available

Site 367

Jerome Laurent, MD

CCPPRB de Paris-Cochin

Hopital TARNIER-COCHIN

89, rue d'Assas

75006, Paris, France

Chair: not available

Site 369

Laurence Bouillet, MD

CCPPRB de Paris-Cochin

Hopital TARNIER-COCHIN

89, rue d'Assas

75006, Paris, France

Chair: not available

Site 371

Elias Toubi, MD

Helsinki Committee

Bnai-Zion Medical Center

47 Golomb St., POB 4940

Haifa 31048, Israel

Chair: not available

Site 372

Avner Reshef, MD

Helsinki Committee

The Chaim Sheba Medical Center

Tel-Hashomer 52621, Israel

Chair: not available

Site 373

Massimo Triggiani, MD, PhD

Comitato Etico per le attivita biomediche

dell'Universita degli Studi di Napoli

Federico II

Via Pansini, 5 - ed.20

80131, Naples, Italy

Chair: not available

Site 374

Stephen Jolles, PhD, MRCP

East London and the City Research Ethics Committee 1

3rd Floor

Aneurin Bevan House

81 Commercial Road

London E1 1RD, U.K.

Chair: not available

**EDEMA3®RD**

Site 301

L. Henry Li, MD

Western Institutional Review Board

3535 Seventh Avenue, SW—Olympia, WA 98502-5010

PO Box 12029—Olympia, WA 98508-2029

Chair: Theodore D. Schultz, JD

Site 302

David Hurewitz, MD

Western Institutional Review Board

3535 Seventh Avenue, SW—Olympia, WA 98502-5010

PO Box 12029—Olympia, WA 98508-2029

Chair: Theodore D. Schultz, JD

Site 303

William Lumry, MD

Western Institutional Review Board

3535 Seventh Avenue, SW—Olympia, WA 98502-5010

PO Box 12029—Olympia, WA 98508-2029

Chair: Theodore D. Schultz, JD

Site 304

Don McNeil, MD

Western Institutional Review Board

3535 Seventh Avenue, SW—Olympia, WA 98502-5010

PO Box 12029—Olympia, WA 98508-2029

Chair: Theodore D. Schultz, JD

Site 305

Jeffrey Leflein, MD

St. Joseph Mercy Health System Clinical Research Committee

5301 East Huron River Drive

PO Box 995 Ann Arbor, MI 48106-0995

Chair: James Mitchiner, MD

Site 306

Lisa Wheatley

Western Institutional Review Board

3535 Seventh Avenue, SW—Olympia, WA 98502-5010

PO Box 12029—Olympia, WA 98508-2029

Chair: Theodore D. Schultz, JD

Site 307

William McCann, MD

Donald Russell, MD

Western Institutional Review Board

3535 Seventh Avenue, SW—Olympia, WA 98502-5010

PO Box 12029—Olympia, WA 98508-2029

Chair: Theodore D. Schultz, JD

Site 308

Stacie Jones, MD

UAMS Institutional Review Board

4301 W. Markham St., Slot 636, Little Rock, AR 72205

Chair: Jimmie Valentine, PhD

Site 309

Karl Sitz, MD

Western Institutional Review Board

3535 Seventh Avenue, SW—Olympia, WA 98502-5010

PO Box 12029—Olympia, WA 98508-2029

Chair: Theodore D. Schultz, JD

Site 310

Timothy Craig, MD

Penn State College of Medicine,

Milton S. Hershey Medical Center

500 University Drive, PO Box 850, Hershey, PA 17033

Chair: Kevin Gleeson, MD

Site 311

Mark Neustrom, MD

Western Institutional Review Board

3535 Seventh Avenue, SW—Olympia, WA 98502-5010

PO Box 12029—Olympia, WA 98508-2029

Chair: Theodore D. Schultz, JD

Site 312

Vincent Bonagura, MD

Biomedical Research Alliance of New York, LLC

Institutional Review Board

225 Community Drive, Suite 100, Great Neck, NY 11021

Chair: Keith Krasinski, MD

Site 313

Mark Davis-Lorton, MD

Western Institutional Review Board

3535 Seventh Avenue, SW—Olympia, WA 98502-5010

PO Box 12029—Olympia, WA 98508-2029

Chair: Theodore D. Schultz, JD

Site 314

Usha Sunkara, MD

Alta Bates Institutional Review Board

2450 Ashby Ave., Berkeley, CA 94705

Chairs: Claire Borkert, MD; James McFeely, MD

Site 315

Arthur Vegh, MD

Western Institutional Review Board

3535 Seventh Avenue, SW—Olympia, WA 98502-5010

PO Box 12029—Olympia, WA 98508-2029

Chair: Theodore D. Schultz, JD

Site 316

Gregory Marcotte, MD

Christiana Care Institutional Review Board

501 West 14th Street, Wilmington, DE 19899

PO Box 1668, Wilmington, DE 19899

Chairs: Robert Cox, MD; Gary Johnson, PhD

Site 317

Albert Sheffer, MD

Brigham's and Women's Hospital Institutional Review Board

Partners Human Research Committee

116 Huntington Ave., Suite 1002, Boston, MA 02115

Site 318

William Yang, MD

Ontario Institutional Review Board

14845-6 Yonge Street, Suite 328, Aurora, ON, Canada L4G 6H8

Chair: Allan Knight

Site 319

Robert Wilkinson, MD

Hawaii Pacific Health Institutional Review board

1100 Ward Avenue, Suite 1045, Honolulu, HI 96814

Chair: not available

Site 320

Duane Wombolt, MD

Western Institutional Review Board

3535 Seventh Avenue, SW—Olympia, WA 98502-5010

PO Box 12029—Olympia, WA 98508-2029

Chair: Theodore D. Schultz, JD

Site 321

Jacques Hebert, MD

IRB Services

45 Wellington St. East, Aurora, Ontario, Canada L4G 1H6

Chair: not available

Site 322

Robert Chrzanowski

Western Institutional Review Board

3535 Seventh Avenue, SW—Olympia, WA 98502-5010

PO Box 12029—Olympia, WA 98508-2029

Chair: Theodore D. Schultz, JD

Site 323

Richard Lockey, MD

Western Institutional Review Board

3535 Seventh Avenue, SW—Olympia, WA 98502-5010

PO Box 12029—Olympia, WA 98508-2029

Chair: Theodore D. Schultz, JD

Site 324

Sai Karlapundi, MD

Western Institutional Review Board

3535 Seventh Avenue, SW—Olympia, WA 98502-5010

PO Box 12029—Olympia, WA 98508-2029

Chair: Theodore D. Schultz, JD

Site 325

Neil Wallen, MD

Western Institutional Review Board

3535 Seventh Avenue, SW—Olympia, WA 98502-5010

PO Box 12029—Olympia, WA 98508-2029

Chair: Theodore D. Schultz, JD

Site 326

Robyn Levy, MD

Western Institutional Review Board

3535 Seventh Avenue, SW—Olympia, WA 98502-5010

PO Box 12029—Olympia, WA 98508-2029

Chair: Theodore D. Schultz, JD

Site 327

Mary Tobin, MD

Rush University Medical Center Institutional Review Board

1725 W. Harrison, Professional Building II, Suite 439, Chicago, IL 60612

Chairs: Allen Korenblit, MD; Howard Kravitz, DO, MPH

Site 328

Gary Kleiner, MD, PhD

Western Institutional Review Board

3535 Seventh Avenue, SW—Olympia, WA 98502-5010

PO Box 12029—Olympia, WA 98508-2029

Chair: Theodore D. Schultz, JD

Site 329

David Elkayam, MD

Western Institutional Review Board

3535 Seventh Avenue, SW—Olympia, WA 98502-5010

PO Box 12029—Olympia, WA 98508-2029

Chair: Theodore D. Schultz, JD

Site 331

Aziza Shad, MD

Georgetown University Institutional Review Board

SW 104 Medical Dental Building

3900 Reservoir Rd. NW, Washington, DC 20057

Chair: Mary Young, MD

Site 332

Arye Rubenstein, MD

Biomedical Research Alliance of New York, LLC

Institutional Review Board

225 Community Drive, Suite 100, Great Neck, NY 11021

Chair Keith Kransinski, MD

Site 333

Eric Schenkel, MD

Western Institutional Review Board

3535 Seventh Avenue, SW—Olympia, WA 98502-5010

PO Box 12029—Olympia, WA 98508-2029

Chair: Theodore D. Schultz, JD

Site 334

David Huston

Institutional Review Board

Baylor College of Medicine

One Baylor Plaza, Suite 600D, Houston, TX 77030

Chair: not available

Site 335

James Baker, MD

Western Institutional Review Board

3535 Seventh Avenue, SW—Olympia, WA 98502-5010

PO Box 12029—Olympia, WA 98508-2029

Chair: Theodore D. Schultz, JD

Site 337

Andrew MacGinnitie, MD

University of Pittsburgh Institutional Review Board

3500 Fifth Avenue, Ground Level, Pittsburgh, PA 15213

Chair: Richard Guido, MD

Site 360

Paul de Buysscher, MD, PhD

Ethische Comissie - Campus Eeklo

vzw 'Elisabeth Zeikenhuis - Heilig Hartkliniek'

Moeie 18 - 9900 Eeklo, Belgium

Chair: Vincent Vandevelde

Site 361

Shmuel Kivity, MSc, MD

Helsinki Committee

Tel Aviv Sourasky Medical Center

Sackler Faculty of Medicine, Tel Aviv University

6 Weizman St., Tel Aviv 64239, Israel

Chair: not available

Site 365

Marco Cicardi, MD

Comitato Etico Indipendente dell'Ospedale San Giuseppe

Ordine ospedaliero di San Giovanni de DIO

Fatebenefratelli di Milano

Via San Vittore, 12

20123, Milan, Italy

Chair: not available

Site 367

Jerome Laurent, MD

CCPPRB de Paris-Cochin

Hopital TARNIER-COCHIN

89, rue d'Assas

75006, Paris, France

Chair: not available

Site 369

Laurence Bouillet, MD

CCPPRB de Paris-Cochin

Hopital TARNIER-COCHIN

89, rue d'Assas

75006, Paris, France

Chair: not available

Site 371

Elias Toubi, MD

Helsinki Committee

Bnai-Zion Medical Center

47 Golomb St., POB 4940

Haifa 31048, Israel

Chair: not available

Site 372

Avner Reshef, MD

Helsinki Committee

The Chaim Sheba Medical Center

Tel-Hashomer 52621, Israel

Chair: not available

Site 373

Massimo Triggiani, MD, PhD

Comitato Etico per le attivita biomediche

dell'Universita degli Studi di Napoli

Federico II

Via Pansini, 5 - ed.20

80131, Naples, Italy

Chair: not available

Site 374

Stephen Jolles, PhD, MRCP

East London and the City Research Ethics Committee 1

3rd Floor

Aneurin Bevan House

81 Commercial Road

London E1 1RD

Chair: not available

**EDEMA4®**

Site 401

Haumin Henry Li, MD, PhD

Western Institutional Review Board

3535 Seventh Avenue, SW

Olympia, WA 98502-5010

Chair: Theodore D. Schultz, JD

Site 403

William Lumry, MD

Western Institutional Review Board

3535 Seventh Avenue, SW

Olympia, WA 98502-5010

Chair: Theodore D. Schultz, JD

Site 404

Donald McNeil, MD

Western Institutional Review Board

3535 Seventh Avenue, SW

Olympia, WA 98502-5010

Chair: Theodore D. Schultz, JD

Site 405

Jeffrey G. Leflein, MD

St. Joseph Mercy Health System Clinical Research Committee

5301 East Huron River Drive

PO Box 995 Ann Arbor, MI 48106-0995

Chair: James Mitchiner, MD

Site 407

Donald Russell, MD and William A. McCann, MD

Western Institutional Review Board

3535 Seventh Avenue, SW

Olympia, WA 98502-5010

Chair: Theodore D. Schultz, JD

Site 408

Stacie Jones, MD

UAMS Institutional Review Board

4301 W. Markham St., Slot 636, Little Rock, AR 72205

Chair: Jimmie Valentine, PhD

Site 409

Karl Sitz, MD

Western Institutional Review Board

3535 Seventh Avenue, SW

Olympia, WA 98502-5010

Chair: Theodore D. Schultz, JD

Site 410

Timothy Craig, DO

Human Subjects Protection Office/Institutional Review Board

Penn State Milton S Hershey Medical Center

600 Centerview Drive MC: A115

Hershey, Pa 17033

Site 411

Mark Neustrom, MD

Western Institutional Review Board

3535 Seventh Avenue, SW

Olympia, WA 98502-5010

Chair: Theodore D. Schultz, JD

Site 413

Mark Davis-Lorton, MD

Western Institutional Review Board

3535 Seventh Avenue, SW

Olympia, WA 98502-5010

Chair: Theodore D. Schultz, JD

Site 414

Usha Sunkara, MD

Alta Bates Institutional Review Board

2450 Ashby Ave.

Berkeley, CA 94705

Chairs: Claire Borkert, MD; James McFeely, MD

Site 415

Arthur B. Vegh, MD

Western Institutional Review Board

3535 Seventh Avenue, SW

Olympia, WA 98502-5010

Chair: Theodore D. Schultz, JD

Site 416
Gregory Marcotte, MD

Christiana Care Institutional Review Board

501 West 14th Street, Wilmington, DE 19899

PO Box 1668

Wilmington, DE 19899

Chairs: Robert Cox, MD; Gary Johnson, PhD

Site 417

Albert Sheffer, MD

Brigham's and Women's Hospital Institutional Review Board

Partners Human Research Committee

116 Huntington Ave., Suite 1002

Boston, MA 02115

Chair: not available

Site 420

Duane Wombolt, MD

Western Institutional Review Board

3535 Seventh Avenue, SW

Olympia, WA 98502-5010

Chair: Theodore D. Schultz, JD

Site 422

Robert Chrzanowski, MD

Western Institutional Review Board

3535 Seventh Avenue, SW

Olympia, WA 98502-5010

Chair: Theodore D. Schultz, JD

Site 423

Richard Lockey, MD

University of South Florida Office of Research

Division of Research Integrity and Compliance

12901 Bruce B. Downs Blvd.

MDC35

Tampa, FL 33612-4799

Site 424

Sai Karlapudi, MD

Western Institutional Review Board

3535 Seventh Avenue, SW

Olympia, WA 98502-5010

Chair: Theodore D. Schultz, JD

Site 425

Neil Wallen, MD

Western Institutional Review Board

3535 Seventh Avenue, SW

Olympia, WA 98502-5010

Chair: Theodore D. Schultz, JD

Site 426

Robyn Levy, MD

Western Institutional Review Board

3535 Seventh Avenue, SW

Olympia, WA 98502-5010

Chair: Theodore D. Schultz, JD

Site 427

Mary Tobin, MD

Office of Research Affairs

Rush University Medical Center

1653 W. Congress Parkway

Chicago, IL 60612

Site 428

Gary Kleiner, MD, PhD

Western Institutional Review Board

3535 Seventh Avenue, SW

Olympia, WA 98502-5010

Chair: Theodore D. Schultz, JD

Site 431

Aziza Shad, MD

Medstar Research Institute-Georgetown University institutional Review Board

Med-Dent Building, SW 104

3900 Reservoir Road, NW

Washington DC 20057-2197

Site 433

Eric Schenkel, MD

Western Institutional Review Board

3535 Seventh Avenue, SW

Olympia, WA 98502-5010

Chair: Theodore D. Schultz, JD

Site 434

David Huston, MD

Institutional Review Board for Baylor College of Medicine and Affiliated

Hospitals

Baylor College of Medicine

Office of Research

One Baylor Plaza, 600D, Houston, TX 77030

Chair: Vernon R. Sutton, MD

Site 437

Andrew MacGinnitie, MD

Univeristy of Pittsburgh Institutional Review Board

3500 Fifth Avenue, Ground Level, Pittsburgh, PA 15213

Chair: Richard Guido, MD

Site 438

Gerald Gleich, MD

University of Utah IRB

Building 512, Room 140

75 S. 2000 E.

Salt Lake City, Utah 84112

Chair: not available

Site 439

Jonathan Bernstein, MD

University of Cincinnati Institutional Review Board

G-08 Wherry Hall, ML#0567

Eden Avenue and Albert Sabin Way

Cincinnati, Ohio 45267-0567

Chair: not available

Site 440

J. Andrew Grant, MD

University of Texas Medical Branch

301 University Blvd, Rebecca Sealy 4.400 (East)

Galveston, TX 77555-0156

Chair: not available

Site 441

Leonard Bielory, MD

Western Institutional Review Board

3535 Seventh Avenue, SW

Olympia, WA 98502-5010

Chair: Theodore D. Schultz, JD

Site 442

Jim Christensen, MD

Western Institutional Review Board

3535 Seventh Avenue, SW

Olympia, WA 98502-5010

Chair: Theodore D. Schultz, JD

Site 443

Marc Riedl, MD

Office for Protection of Research Subjects (OPRS)

11000 Kinross Avenue, Suite 102

Box 951694

Los Angeles, California 90095-1694

Chair: not available

Site 445

Keith Paull, MD

Western Institutional Review Board

3535 Seventh Avenue, SW

Olympia, WA 98502-5010

Chair: Theodore D. Schultz, JD

Site 448

Mustafa Shennak, MD

Triumpharma LLC, IRB

PO Box 2233

Al-Yarooty Street

Amman, 1194, Jordan

Jordan University Hospital IRB

Queen Rania Street

Amman, Jordan

Site 449

William Yang, MD

IRB Services

372 Hollandview Trail

Suite 300

Aurora, Ontario L46 OA5

Canada

Site 451

Gordon Sussman, MD

IRB Services

372 Hollandview Trail

Suite 300

Aurora, Ontario L46 OA5

Canada

Site 452

John Cohn, MD

Western Institutional Review Board

3535 Seventh Avenue, SW

Olympia, WA 98502-5010

Chair: Theodore D. Schultz, JD

Site 453

Aaron Davis, MD

Western Institutional Review Board

3535 Seventh Avenue, SW

Olympia, WA 98502-5010

Chair: Theodore D. Schultz, JD

Site 454

Jacob Offenberger, MD

Western Institutional Review Board

3535 Seventh Avenue, SW

Olympia, WA 98502-5010

Chair: Theodore D. Schultz, JD

Site 456

Daniel Soteres, MD, MPH

Western Institutional Review Board

3535 Seventh Avenue, SW

Olympia, WA 98502-5010

Chair: Theodore D. Schultz, JD

Site 457

Jeffrey Bruner, DO

Western Institutional Review Board

3535 Seventh Avenue, SW

Olympia, WA 98502-5010

Chair: Theodore D. Schultz, JD

Site 458

Mary Beth Hogan, MD

University of Nevada Reno Office of Human Protection

University of Nevada, Reno

205 Ross Hall/331

Reno, Nevada 89557

Chair: not available

Site 459

Michael Keslin, MD

Western Institutional Review Board

3535 Seventh Avenue, SW

Olympia, WA 98502-5010

Chair: Theodore D. Schultz, JD

Site 460

Christopher Chang, MD

Western Institutional Review Board

3535 Seventh Avenue, SW

Olympia, WA 98502-5010

Chair: Theodore D. Schultz, JD

Site 461

Clive Roberson, MD

Western Institutional Review Board

3535 Seventh Avenue, SW

Olympia, WA 98502-5010

Chair: Theodore D. Schultz, JD

**DX-88/19**

Site 401

Huamin Henry Li, MD, PhD

Western Institutional Review Board

3535 Seventh Avenue, SW

Olympia, WA 98502-5010

Chair: Theodore D. Schultz, JD

Site 403

William Lumry, MD

Western Institutional Review Board

3535 Seventh Avenue, SW

Olympia, WA 98502-5010

Chair: Theodore D. Schultz, JD

Site 404

Donald McNeil, MD

Western Institutional Review Board

3535 Seventh Avenue, SW

Olympia, WA 98502-5010

Chair: Theodore D. Schultz, JD

Site 405

Jeffrey G. Leflein, MD

St. Joseph Mercy Health System Institutional Review Board

5301 East Huron River Drive

PO Box 995 Ann Arbor, MI 48106-0995

Chair: James Mitchner, MD

Site 407

William A. McCann, MD

Western Institutional Review Board

3535 Seventh Avenue, SW

Olympia, WA 98502-5010

Chair: Theodore D. Schultz, JD

Site 408

Stacie Jones, MD

University of Arkansas for Medical Sciences

UAMS Institutional Review Board

4301 W. Markham St., Slot 636, Little Rock, AR 72205

Chairs: Louanne Lawson, PhD, Kathleen Eisenach, PhD, Mahendran Mahadevan, PhD

Site 409

Karl Sitz, MD

Western Institutional Review Board

3535 Seventh Avenue, SW

Olympia, WA 98502-5010

Chair: Theodore D. Schultz, JD

Site 410

Timothy Craig, DO

Human Subjects Protection Office/Institutional Review Board

Penn State Milton S Hershey Medical Center

600 Centerview Drive MC: A115, PO 885

Hershey, Pa 17033

Chair: Kevin Gleeson, M.D.

Site 411

Mark Neustrom, MD

Western Institutional Review Board

3535 Seventh Avenue, SW

Olympia, WA 98502-5010

Chair: Theodore D. Schultz, JD

Site 413

Mark Davis-Lorton, MD

Western Institutional Review Board

3535 Seventh Avenue, SW

Olympia, WA 98502-5010

Chair: Theodore D. Schultz, JD

Site 414

Usha Sunkara, MD

Alta Bates Institutional Review Board

2450 Ashby Ave.

Berkeley, CA 94705

Chairs: Claire Borkert, MD; James McFeely, MD

Site 415

Arthur B. Vegh, MD

Western Institutional Review Board

3535 Seventh Avenue, SW

Olympia, WA 98502-5010

Chair: Theodore D. Schultz, JD

Site 416

Gregory Marcotte, MD

Christiana Care Institutional Review Board

501 West 14th Street, Wilmington, DE 19899

PO Box 1668

Wilmington, DE 19899

Chairs: Robert Cox, MD; Gary Johnson, PhD

Site 417

Albert Sheffer, MD

Brigham's and Women's Hospital Institutional Review Board

Partners Human Research Committee

116 Huntington Ave., Suite 1002

Boston, MA 02115

Chair: N/A per policy

Site 420

Duane Wombolt, MD

Western Institutional Review Board

3535 Seventh Avenue, SW

Olympia, WA 98502-5010

Chair: Theodore D. Schultz, JD

Site 422

Robert Chrzanowski, MD

Western Institutional Review Board

3535 Seventh Avenue, SW

Olympia, WA 98502-5010

Chair: Theodore D. Schultz, JD

Site 423

Richard Lockey, MD

Western Institutional Review Board

3535 Seventh Avenue, SW

Olympia, WA 98502-5010

Chair: Theodore D. Schultz, JD

Site 424

Sai Karlapudi, MD

Western Institutional Review Board

3535 Seventh Avenue, SW

Olympia, WA 98502-5010

Chair: Theodore D. Schultz, JD

Site 425

Neil Wallen, MD

Western Institutional Review Board

3535 Seventh Avenue, SW

Olympia, WA 98502-5010

Chair: Theodore D. Schultz, JD

Site 426

Robyn Levy, MD

Western Institutional Review Board

3535 Seventh Avenue, SW

Olympia, WA 98502-5010

Chair: Theodore D. Schultz, JD

Site 427

Mary Tobin, MD

Rush University Medical Center

Research and Clinical Trials Administration Office

1653 W. Congress Parkway

Chicago, IL 60612

Chairs: Allen Korenblit, MD and Howard Kravitz, DO

Site 428

Gary Kleiner, MD, PhD

Western Institutional Review Board

3535 Seventh Avenue, SW

Olympia, WA 98502-5010

Chair: Theodore D. Schultz, JD

Site 431

Aziza Shad, MD

Medstar Research Institute-Georgetown University institutional Review Board

Med-Dent Building, SW 104

3900 Reservoir Road, NW

Washington DC 20057-2197

Chair: Korin Hudson, MD

Site 433

Eric Schenkel, MD

Western Institutional Review Board

3535 Seventh Avenue, SW

Olympia, WA 98502-5010

Chair: Theodore D. Schultz, JD

Site 434

David Huston, MD

Texas A&M University

Human Subjects Protection Program Office

General Services Complex

750 Agronomy Rd., Suite 3501

TAMU 1186 (mailstop_

College Station, TX 77843-1186

Chairs: Dr. J. Stephen Moore, Ms. Mary Elizabeth Herring

Site 437

Andrew MacGinnitie, MD

University of Pittsburgh Institutional Review Board

3500 Fifth Avenue, Suites 105 & 106, Pittsburgh, PA 15213

Chair: Richard Guido, MD

Site 438

Gerald Gleich, MD

University of Utah IRB

Building 512, Room 140

75 S. 2000 E.

Salt Lake City, Utah 84112

Chairs: Gerald Treiman MD

Site 439

Jonathan Bernstein, MD

University of Cincinnati Institutional Review Board

G-08 Wherry Hall, PO Box 670567

3225 Eden Ave

Cincinnati, Ohio 45267-0567

Chair: Michael Linke, PhD

Site 440

J. Andrew Grant, MD

University of Texas Medical Branch

Office of Research Subjects Protection

Institutional Review Board

4500 Rebecca Sealy Hospital

301 University Blvd.

Galveston, TX 77555-0158

Chair: Radoslaw Bukowski, MD; Ivan Kessel, MD; David Weigle, PhD

Site 441

Leonard Bielory, MD

Western Institutional Review Board

3535 Seventh Avenue, SW

Olympia, WA 98502-5010

Chair: Theodore D. Schultz, JD

Site 442

Jim Christensen, MD

Western Institutional Review Board

3535 Seventh Avenue, SW

Olympia, WA 98502-5010

Chair: Theodore D. Schultz, JD

Site 443

Marc Riedl, MD

Office for Protection of Research Subjects (OPRS)

11000 Kinross Avenue, Suite 102

Box 951694

Los Angeles, California 90095-1694

Chair: Lawrence Wolinsky, Daniel Clemens, James McGough,

Site 445

Keith Paull, MD

Western Institutional Review Board

3535 Seventh Avenue, SW

Olympia, WA 98502-5010

Chair: Theodore D. Schultz, JD

Site 448

Mustafa Shennak, MD

Triumpharma LLC, IRB

PO Box 2233

Al-Yarooty Street

Amman, 1194, Jordan

Chair: N/A

Site 449

William Yang, MD

IRB Services

372 Hollandview Trail

Suite 300

Aurora, Ontario L4G OA5

Canada

Chair: Valia Lestou, PhD; Stephen Hoption Cann, PhD

Site 451

Gordon Sussman, MD

IRB Services

372 Hollandview Trail

Suite 300

Aurora, Ontario L4G OA5

Canada

Chair: Valia Lestou, PhD; Stephen Hoption Cann, PhD

Site 452

John Cohn, MD

Western Institutional Review Board

3535 Seventh Avenue, SW

Olympia, WA 98502-5010

Chair: Theodore D. Schultz, JD

Site 453

Aaron Davis, MD

Western Institutional Review Board

3535 Seventh Avenue, SW

Olympia, WA 98502-5010

Chair: Theodore D. Schultz, JD

Site 454

Jacob Offenberger, MD

Western Institutional Review Board

3535 Seventh Avenue, SW

Olympia, WA 98502-5010

Chair: Theodore D. Schultz, JD

Site 456

Daniel Soteres, MD, MPH

Western Institutional Review Board

3535 Seventh Avenue, SW

Olympia, WA 98502-5010

Chair: Theodore D. Schultz, JD

Site 457

Jeffrey Bruner, DO

Western Institutional Review Board

3535 Seventh Avenue, SW

Olympia, WA 98502-5010

Chair: Theodore D. Schultz, JD

Site 458

Mary Beth Hogan, MD

Western Institutional Review Board

3535 Seventh Avenue, SW

Olympia, WA 98502-5010

Chair: Theodore D. Schultz, JD

Site 459

Michael Keslin, MD

Western Institutional Review Board

3535 Seventh Avenue, SW

Olympia, WA 98502-5010

Chair: Theodore D. Schultz, JD

Site 460

Christopher Chang, MD

Western Institutional Review Board

3535 Seventh Avenue, SW

Olympia, WA 98502-5010

Chair: Theodore D. Schultz, JD

Site 461

Clive Roberson, MD

Western Institutional Review Board

3535 Seventh Avenue, SW

Olympia, WA 98502-5010

Chair: Theodore D. Schultz, JD
